# Non-Cell-Autonomous Factors Implicated in Parvalbumin Interneuron Maturation and Critical Periods

**DOI:** 10.3389/fncir.2022.875873

**Published:** 2022-04-26

**Authors:** Rachel Gibel-Russo, David Benacom, Ariel A. Di Nardo

**Affiliations:** Centre for Interdisciplinary Research in Biology (CIRB), Collège de France, CNRS, INSERM, Labex MemoLife, PSL Research University, Paris, France

**Keywords:** parvalbumin (PV), homeoprotein, astrocyctes, oligodedrocytes, microglia, epigenetics, perineuronal net (PNN)

## Abstract

From birth to adolescence, the brain adapts to its environmental stimuli through structural and functional remodeling of neural circuits during critical periods of heightened plasticity. They occur across modalities for proper sensory, motor, linguistic, and cognitive development. If they are disrupted by early-life adverse experiences or genetic deficiencies, lasting consequences include behavioral changes, physiological and cognitive deficits, or psychiatric illness. Critical period timing is orchestrated not only by appropriate neural activity but also by a multitude of signals that participate in the maturation of fast-spiking parvalbumin interneurons and the consolidation of neural circuits. In this review, we describe the various signaling factors that initiate critical period onset, such as BDNF, SPARCL1, or OTX2, which originate either from local neurons or glial cells or from extracortical sources such as the choroid plexus. Critical period closure is established by signals that modulate extracellular matrix and myelination, while timing and plasticity can also be influenced by circadian rhythms and by hormones and corticosteroids that affect brain oxidative stress levels or immune response. Molecular outcomes include lasting epigenetic changes which themselves can be considered signals that shape downstream cross-modal critical periods. Comprehensive knowledge of how these signals and signaling factors interplay to influence neural mechanisms will help provide an inclusive perspective on the effects of early adversity and developmental defects that permanently change perception and behavior.

## Introduction

Critical periods (CPs) of heightened plasticity shape neural circuits according to experience during postnatal brain development (Reh et al., 2020). These distinct plasticity windows occur not only across primary sensory areas, such as the primary visual and auditory cortices but also in multimodal areas, such as the insular cortex and the medial prefrontal cortex (mPFC; Testa-Silva et al., 2012; Gogolla et al., 2014). Separate CPs can occur at the same time in different brain areas, but complex functions may depend on the closure of an upstream “primary” CP and thus require sequential CPs (Nakamura et al., 2020). CP timing is driven by the maturation of fast-spiking (FS) inhibitory interneurons that express parvalbumin (PV). While PV cells are located throughout the brain, FS-PV cells residing in supragranular cortical layers drive CP onset and circuit rewiring (Fagiolini et al., 2004). They receive excitatory input from local pyramidal cells as well as long-range inputs from the thalamus and hippocampus (Faini et al., 2018; Yang et al., 2021), and they receive inhibitory inputs from themselves (autapses), from other FS-PV cells, and from SST, VIP, and CCK interneurons (Méndez and Bacci, 2011). They also connect with dopaminergic, serotonergic, and cholinergic fibers (Sun et al., 2019). The physiological maturation of FS-PV cells results in strong inhibitory output on the soma or the axon initial segment of nearby pyramidal cells, which provides control of excitatory currents, alters the excitatory–inhibitory (E/I) balance, permits large-scale changes in neural circuitry, and influences rhythmic oscillations (Sohal et al., 2009; Hu et al., 2014).

As FS-PV cells mature, they become enwrapped by perineuronal nets (PNNs) composed of glycans, proteoglycans, and proteins originating from either FS-PV cells or surrounding cells. PNNs are a condensed extracellular matrix (ECM) providing specific electrical properties and a specialized micro-environment that stabilizes synapses and attracts non-cell-autonomous factors (for reviews, see Testa et al., 2019; Carulli and Verhaagen, 2021). Precise PNN accumulation, in terms of both composition and timing, is implicated in CP onset but plays a major role as a molecular brake limiting structural plasticity for CP closure (for review, see Fawcett et al., 2019). Removal of PNNs can allow for functional plasticity in adult rodents (Pizzorusso et al., 2002; Beurdeley et al., 2012).

**Figure 1:**
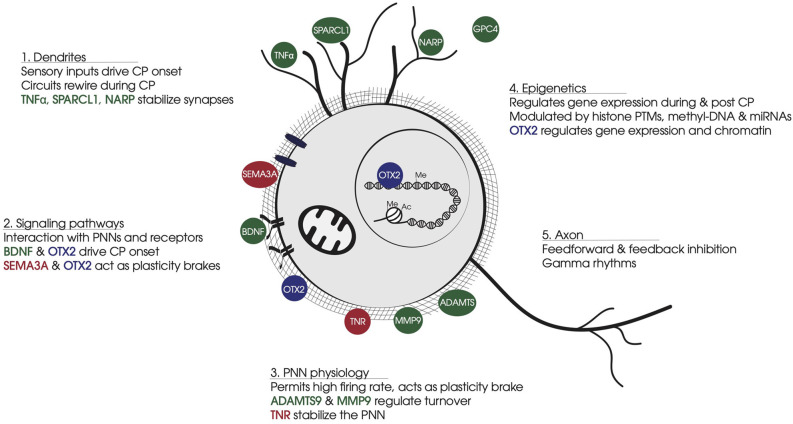
Summary of non-cell-autonomous factors involved in FS-PV cell physiology. **(1)** Dendrites: Non-cell-autonomous factors stabilize synapse number and strength of sensory inputs that drive CP onset and FS-PV cell synapse maturation. **(2)** Signaling pathways: Non-cell-autonomous factors bind specific receptors or glycosaminoglycan motifs in PNNs. BDNF-TRKB triggers activity-dependent pathways. OTX2 binding to CSPGs leads to cell internalization and translocation to the nucleus. SEMA3A interacts with CSPGs and stabilizes synapses on FS-PV cell soma. **(3)** PNN physiology: PNNs develop around FS-PV cells during CP, providing neuroprotection against oxidative damage induced by the fast-spiking activity of FS-PV cells. PNNs stabilize neural networks and limit synapses formation. Secreted ECM enzymes provide turnover for PNN dynamics, while other non-cell-autonomous factors participate in PNN stabilization. **(4)** Epigenetics: During the CP, the FS-PV cell transcriptome is regulated in response to the cellular environment *via* histone PTMs and DNA methylation. OTX2 affects gene expression and chromatin conformation in part through transcription regulation *Gadd45b/g*. **(5)** Axon: FS-PV cell outputs on principal cells generate gamma rhythms. Non-cell-autonomous factors participate in axon myelination and the stabilization of pre-synaptic receptors. Color code: In green, non-cell-autonomous factors contributing to CP onset; in red, non-cell-autonomous factors contributing to CP closure; in blue, non-cell-autonomous factors contributing in both opening and closure of CP. Abbreviations: CP, critical period; PNN, perineuronal net; FS-PV, fast-spiking-parvalbumin; CSPG, chondroitin sulfate proteoglycans; PTM, post-translational modification.

Non-cell-autonomous factors (summarized in Figure 1) also shape the connectivity and the electrical properties of FS-PV cells during CPs and determine the extent to which the environment—local, systemic, and external—can affect cortical circuit dynamics throughout life. A precise and detailed understanding of such factors will help identify potential modulators of plasticity in both childhood and adulthood in order to reverse or repair brain disorders and trauma.

## Non-Cell-Autonomous Factors from Local Cells for Short-Range Interactions

### Cellular Sources

Short-range non-cell-autonomous proteins that affect FS-PV cell function come from surrounding neurons, astrocytes, microglia, and oligodendrocytes. While these cells can interact and exchange factors within the synaptic compartment to guide synapse formation and function, they also secrete factors that signal through the ECM (Table 1). All of these cell types secrete neurotrophic factors, proteases, and PNN components, while astrocytes and microglia also release proteins that shape synaptic function. Astrocytes participate in synapse formation, connectivity, transmission, and plasticity by regulating the extracellular ionic environment and recycling neurotransmitters (for review, see Santello et al., 2019), and by also secreting proteoglycans (for review, see Wiese et al., 2012). Microglia respond to changes in neural activity and help shape circuitry through various mechanisms including phagocytosis or through the secretion of active peptides and enzymes (for review, see Salter and Beggs, 2014). Both astrocytes and microglia have recently been implicated as active participants in CP plasticity (Sipe et al., 2016; Kalish et al., 2020; Ackerman et al., 2021; Ribot et al., 2021). Beyond secreting some of the above factors, oligodendrocytes provide myelination, which initially coincides with CP opening in the sensory cortices but ultimately participates as an important molecular brake for CP closure (for review, see Fletcher et al., 2021). In order to focus on more widely dispersed factors, we chose to not elaborate further on neurotransmitters, synaptic pruning, adhesion molecules, and myelination.

**Table 1:** Sources, targets, and functions of non-cell-autonomous molecules affecting FS-PV cell activity.

**Source**	**Molecule**	**FS-PV cell target**	**Function**	**Ref.**
**Neurons**	ADAMTS-8/-15	ECM	ECM remodeling, Synapse reorganization	**1, 2**
	BDNF	TRKB	Maturation of GABAergic circuit, Synaptic transmission and plasticity, CP onset	**3**
	NARP	AMPAR	AMPAR clustering	**4**
	MMP2/9	ECM	Functional plasticity, ECM remodeling, Synapse reorganization	**5**
	TNR	CSPG	ECM assembly	**6**
	tPA	BDNF	Conversion of pro-BDNF into m-BDNF	**7, 8**
**Astrocytes**	ADAMTS-1/-4/-5/-9	ECM	Functional plasticity, ECM remodeling, Synapse reorganization	**1,9**
	BDNF	TRKB	Putative (see Neurons)	**10**
	CSPG	ECM	ECM remodeling	**11**
	GPC4	RPTP	Synapse maturation	**12**
	MMP9	ECM	Functional plasticity, ECM remodeling, Synapse reorganization	**5,13**
	SPARCL1	NRX1, NRGL1	Synapse formation	**14, 15**
	tPA	BDNF	tPA recycling	**16**
**Microglia**	BDNF	TRKB	Putative (see Neurons)	**10, 17**
	Cathepsin-S	CSPG	ECM remodeling	**18**
	MMP9	ECM	Functional plasticity, ECM remodeling, Synapse reorganization	**5,19**
**Oligodendrocytes**	ADAMTS-4	ECM	ECM remodeling	**2**
	BDNF	TRKB	Putative (see Neurons)	**10, 20**
	MMP9	ECM	Functional plasticity, ECM remodeling, Synapse reorganization	**5**
	TNR	CSPG	ECM assembly	**6**
	tPA	BDNF	Putative (see Neurons)	**8**

*Abbreviations: CSPG, chondroitin sulfate proteoglycan; CP, critical period; ECM, extracellular matrix; FS-PV, fast-spiking-parvalbumin. References: 1. Rossier et al. (2015); 2. Levy et al. (2015); 3. Chao (2003); 4. Chang et al. (2010); 5. Reinhard et al. (2015); 6. Carulli et al. (2006); 7. Mataga et al. (2002); 8. Louessard et al. (2016); 9. Lemarchant et al. (2013); 10. Dougherty et al. (2000); 11. Wiese et al. (2012); 12. Dowling and Allen (2018); 13. Ribot et al. (2021); 14. Bradshaw (2012); 15. Singh et al. (2016); 16. Casse et al. (2012); 17. Parkhurst et al. (2013); 18. Pantazopoulos et al. (2020); 19. Venturino et al. (2021); 20. Bagayogo and Dreyfus (2009)*.

### Growth Factors

One of the first non-cell-autonomous molecules discovered to be involved in CPs was brain-derived neurotrophic factor (BDNF), which signals through interaction with its canonical receptor TRKB on FS-PV cells. BDNF is typically secreted as pro-BDNF which is generally cleaved by plasmin to release BDNF (Lu et al., 2005) that then activates TRKB phosphorylation to initiate intracellular cascades of early/immediate-genes leading ultimately to FS-PV cell maturation for CP plasticity onset (Huang et al., 1999). Loss of function studies of TRKB receptors in FS-PV cells demonstrate global changes in network connectivity, as well as behavioral, autistic-like phenotypes (Xenos et al., 2018), probably caused by brain-wide dysregulation of CPs. The sensitivity of FS-PV cells to BDNF may be dampened by PNNs, which activate protein tyrosine phosphatase sigma (PTPσ) receptors (Lesnikova et al., 2021). When bound to chondroitin sulfate proteoglycan (CSPG), PTPσ promotes TRKB dephosphorylation to abolish signaling and limit BDNF-induced plasticity in FS-PV cells. Thus FS-PV cells are likely less responsive to BDNF after CP closure. Although astrocytes, microglia, and oligodendrocytes also secrete pro-BDNF (Dougherty et al., 2000), these cell types have not been formally shown to affect FS-PV TRKB signaling in cortical CPs. Nevertheless, it remains highly likely that they participate. For example, secretion of BDNF from microglia is critical for learning-dependent synaptic plasticity in the motor cortex (Parkhurst et al., 2013), and affects inhibitory synaptic transmission *via* TrkB signaling in the spinal cord (Coull et al., 2005) and hippocampus (Zheng et al., 2011). Also, oligodendrocytes actively participate in synaptic transmission and plasticity through BDNF signaling in the developing brain (Jang et al., 2019).

Neuregulin-1 (NRG1), a trophic factor containing an epidermal growth factor (EGF) domain, is expressed in astrocytes and in both inhibitory and excitatory cortical neurons, and exists in multiple isoforms including the soluble types I and II (Liu et al., 2011). NRG1 signals through the ErbB tyrosine receptor kinase family and specific NRG1/ErbB4 signaling in FS-PV cells regulate connectivity (Fazzari et al., 2010) and CP plasticity (Gu et al., 2016; Sun et al., 2016). While ErbB4 is expressed by FS-PV cells, it remains unclear whether NRG1 signaling is autocrine or paracrine, given that both FS-PV cells and excitatory neurons can release NRG1 into the extracellular space (Grieco et al., 2020). Interestingly, NRG1 signaling also involves oligodendrocytes that express ErbB3. In the mouse mPFC, NRG1/ErbB3 signaling is essential during a post-weaning CP for social learning which affects oligodendrocyte maturation and lifelong myelination (Makinodan et al., 2012). The NRG1/ErbB4 signal also involves VIP interneurons in the mPFC, and NRG1 is gaining attention as a therapeutic target for psychiatric disorders (Shi and Bergson, 2020).

### Perineuronal Nets

Built with diverse components originating from neurons, astrocytes, and oligodendrocytes, PNNs are lattice-like structures primarily composed of hyaluronan (HA), CSPGs, and cross-linking proteins from the link protein family (HAPLNs) and tenascin-R (TNR; for reviews, see Fawcett et al., 2019; Testa et al., 2019). PNN assembly is activity-dependent (Dityatev et al., 2007), requiring sensory input during postnatal development in order to form (Pizzorusso et al., 2002; McRae et al., 2007; Kind et al., 2013), and their maintenance in adulthood can be regulated by local network activity (Devienne et al., 2021). Physiologically, PNNs act as molecular brakes that progressively decrease plasticity as they condense around FS-PV cell soma and proximal dendrites during the CP, and eventually restrict plasticity in adulthood (Pizzorusso et al., 2002). Through enzymatic removal of PNNs *in vivo*, it has been shown that PNNs can affect FS-PV cell excitability and spontaneous activity (Lensjø et al., 2017; Hayani et al., 2018; Carceller et al., 2020), and selectively dampen thalamic excitation (Faini et al., 2018).

PNNs protect neurons from oxidative stress and toxic proteins (Miyata et al., 2007; Cabungcal et al., 2013; Suttkus et al., 2014), and their polyanionic structure limits the toxicity inherent to the high activity of FS-PV cells by buffering the cations involved in neurotransmission (Brückner et al., 1993; Härtig et al., 1999). They also interact with signaling molecules, such as SEMA3A and OTX2 (described below), and potentially affect the dynamics of membrane-bound proteins. While OTX2 has been shown to regulate the expression of CSPGs within FS-PV cells (Hou et al., 2017; Lee et al., 2017), a complete picture of PNN molecule expression regulation has been difficult to obtain given that their components come from multiple sources. While FS-PV cells express all of the required molecules, including HA and HAPLNs, surrounding glial cells and other neurons can also contribute CSPGs and TNR (Testa et al., 2019). Thus, the assembly of PNNs around FS-PV cells has the potential to be greatly influenced by changes in the expression of neighboring cells. The picture is further complicated by the regulation of PNN structure by multiple metalloproteases (described below) that again come from multiple sources. In fact, although PNNs are often thought of as fixed structures, they remain dynamic in adulthood in response to changes in FS-PV activity and diurnal fluctuations (Pantazopoulos et al., 2020; Devienne et al., 2021; Harkness et al., 2021).

### Proteases

Tissue-type plasminogen activator protein (tPA), originally identified as an anti-clotting agent in the blood, is a major serine protease in the brain that plays several roles in brain plasticity (for review, see Hensch, 2005) and is expressed by neurons and oligodendrocytes (Louessard et al., 2016). Proteolysis by tPA permits experience-dependent spine motility by degrading ECM and cell-adhesion proteins. It converts plasminogen to plasmin, which in turn not only degrades ECM but also activates metalloproteases, chemokines, and neurotrophic factors, such as BDNF. tPA is released by both axons and dendrites through exosomal vesicles, with axonal release being activity-dependent (for review, see Lenoir et al., 2019). Astrocytes also regulate tPA levels, possibly by expressing tPA, but also by recycling the tPA secreted by neurons in the synaptic cleft so that it can then bind to various receptors and act as a neuromodulator (Casse et al., 2012). Permissive amounts of tPA may therefore integrate functional and structural changes downstream of the E/I balance for CP plasticity.

Matrix metalloproteinases (MMPs) are a family of zinc-binding endopeptidases that selectively degrade proteoglycans, growth factors, cytokines, chemokines, myelin-associated proteins, and cell adhesion molecules in the ECM and PNNs (for review, see Huntley, 2012). MMPs can be activated by tPA and, in turn, be removed by tissue inhibitors of metalloproteinases (TIMPs). While several MMPs are found in the brain, MMP9 is the only member known to be secreted by astrocytes, oligodendrocytes, microglia, and neurons (for review, see Reinhard et al., 2015). MMP9 regulates both functional plasticity and ECM remodeling by shaping the pericellular and synaptic environment and by activating signaling molecules. CP closure is accompanied by changes in astrocytic networks that dampen their secretion of MMP9 and favor PNN formation (Ribot et al., 2021). Conversely, MMP9 secreted by microglia can remodel PNNs in adult plasticity paradigms (Venturino et al., 2021). MMP9 knock-out mice show attenuated plasticity during CPs and show subtle alterations in microglia morphology suggesting microglia function is changed (Kelly et al., 2015). In keeping with the possibility that MMPs can also liberate molecules that impede neurite outgrowth, recent evidence shows that inhibition of MMP2 and MMP9 can either limit or promote adult visual cortex plasticity depending on the nature of the insult (Akol et al., 2022).

Other proteases include cathepsin-S and enzymes from the ADAMTS (A Disintegrin and Metalloproteinase with Thrombospondin motifs) family. Cathepsin-S, which is expressed and secreted by microglia throughout the adult brain, is a member of the lysosomal cysteine protease family that can degrade proteoglycans, and was recently shown to digest PNNs in a circadian manner (see below; Pantazopoulos et al., 2020). Similar to MMPs, ADAMTSs are also zinc-binding endopeptidases, but select members expressed in the brain show substrate specificity for aggrecan and other CSPGs within the ECM and the PNNs of FS-PV cells (Kelwick et al., 2015). While they are mainly expressed by astrocytes (Lemarchant et al., 2013), ADAMTS-8 and -15 were found to be expressed in somatosensory FS-PV cells (Rossier et al., 2015), suggesting more widespread expression patterns are possible. Although ADAMTS8 and ADAMTS9 have been shown to be upregulated during CPs (Lee et al., 2017; Apulei et al., 2019), how this family of proteases is regulated remains unknown.

### Glycoproteins

Several secreted glycoproteins, including glypicans (GPC), pentraxin 1 (NPTX1), and neuronal activity-regulated pentraxin (NARP or NPTX2), fall within related pathways that may affect FS-PV cell function. Glypicans are a family of heparan sulfate proteoglycans that are localized to the neuronal membrane *via* glycosylphosphatidylinositol (GPI) anchor and can be released from the cell surface upon cleavage (Filmus et al., 2008). Although not formally implicated in CP regulation, GPC4 is an astrocyte-secreted protein expressed throughout early postnatal development (Dowling and Allen, 2018), and has NARP as a downstream target that regulates FS-PV cells (see below). GPC4 binds to presynaptic type 2a receptor protein tyrosine phosphatases (RPTPs) inducing the release of NPTX1 (Farhy-Tselnicker et al., 2017). NPTX1 makes a heterocomplex with NARP, which binds to AMPA receptors and triggers their clustering in post-synaptic neurons. NARP is an immediate early gene that regulates synaptic strength and is enriched selectively at excitatory synapses impinging on FS-PV cells (Chang et al., 2010). In *Narp^−/−^* mice, inhibition from FS-PV cells is impaired at the onset of ocular dominance CP plasticity (Gu et al., 2013). Interestingly, enzymatic treatment to remove PNNs also removes NARP from the surface of neuronal dendrites (Gu et al., 2013). Conversely, NARP enhances PNN formation (Van’t Spijker et al., 2019), suggesting that there is a feedback interaction between NARP and PNNs. Given that GPCs are expressed in the choroid plexus and secreted into the cerebrospinal fluid (CSF; Lugert et al., 2017; Dani et al., 2021), their involvement in CP regulation may have been missed due to long-range signaling (see below).

In the visual cortex, the synapse-regulating protein SPARCL1 (Hevin) is a glycoprotein secreted by astrocytes and implicated in experience-dependent plasticity. Once secreted, it can participate in the formation of synapses by linking presynaptic Neurexin-1 (NRX1) to postsynaptic Neuroligin 1 (NLGN1), which together regulate synaptic signal transmission (Gan and Südhof, 2020). In the visual cortex, SPARCL1 has been shown to bridge thalamocortical afferents and be required for ocular dominance CP plasticity (Singh et al., 2016; Ribic et al., 2019). SPARCL1 also has a synaptogenic activity that can be inhibited by ADAMTS4 and MMPs (1, 3, and 9), which cleave SPARCL1 to generate a SPARC-like fragment (SLF) that in turn competes with SPARCL1 (Bradshaw, 2012).

Plasticity states and interneuron maturation are also influenced by levels of long polysialic acid (PSA) chains attached to neural cell adhesion molecules (NCAM). PSA-NCAM is expressed by both glial cells and neurons and provides a highly hydrated polymer that lubricates extracellular space and minimizes surface interactions between cells, and the NCAM extracellular region (NCAM-EC) can be released as a soluble fragment (for review, see Rutishauser, 2008). In the juvenile visual cortex, the expression of PSA-NCAM undergoes a dramatic activity-dependent decline that is permissive for the maturation of FS-PV cells (Di Cristo et al., 2007). In the rodent mPFC, PSA-NCAM expression is restricted to interneurons, at least in the adult, and plays a dopamine-dependent permissive role for inhibitory circuit structural plasticity (Castillo-Gómez et al., 2011). Conversely, non-PSA NCAM participates in the removal of interneuron synapses, but this process can be inhibited through NCAM interaction with CSPGs, further highlighting the role of PNNs for synaptic stability (Sullivan et al., 2018). Finally, NCAM-EC has been shown to restrict neurite branching and outgrowth, and its overexpression stunts FS-PV cell maturation in the mPFC (Brennaman and Maness, 2008). Thus, while NCAM is not strictly a non-cell-autonomous factor, its various forms and fragments can impact the ECM and FS-PV cell plasticity, thereby influencing juvenile CPs and adult functions. Indeed, its misexpression is linked with chronic stress and psychiatric disorders (Bueno-Fernandez et al., 2021).

## Systemic and Non-Cell-Autonomous Factors for Long-Range Interactions

### Cellular and Systemic Sources

Brain plasticity can be guided by signals coming from the periphery. Certain molecules in the cortical vasculature can pass the blood-brain barrier (BBB), either through transmembrane diffusion, active transport, or transcytosis (for reviews, see Abbott et al., 2010; Sweeney et al., 2019). Some diffuse through the extracellular space while others are mediated by astrocytic endfeet and local microglia. The choroid plexus is also highly vascularized and provides the blood-cerebrospinal fluid barrier (BCSFB) that is permissively different than the BBB and controls leukocyte entry into the CSF (for reviews, see Redzic, 2011; Ghersi-Egea et al., 2018; Cui et al., 2021). These structures are also responsive to corticosteroids and hormones. Furthermore, the choroid plexus secretes CSF which provides factors that regulate neural function and participates in the clearance of waste metabolites from the extracellular space (for reviews, see Praetorius and Damkier, 2017; Fame and Lehtinen, 2020). Diurnal rhythms also affect choroid plexus function, resulting in altered CSF composition, parenchyma clearance, and brain homeostasis (Myung et al., 2018).

### Circadian Rhythms

Repeated daily patterns in gene expression, physiology, and behavior are driven by self-sustained biological clocks of approximately 24 h (circadian). A feedback loop between the transcription factors CLOCK and BMAL1 drives not only the central diurnal oscillator within the hypothalamic suprachiasmatic nucleus but is also observed in nearly all mammalian tissues (for review, see Lowrey and Takahashi, 2011). The choroid plexus also provides a strong circadian clock component that imparts diurnal changes in CSF composition and production, which affects not only the timing of metabolite clearance in the brain but also the distribution of clock signals to other brain regions (Myung et al., 2018). By using *Clock* knock-out mouse models, it was revealed that circadian-dependent expression of *Per1* and *Dbp* in the primary visual cortex is mediated by the CLOCK:BMAL1 oscillator (Kobayashi et al., 2015). These mice have delayed CP timing, and it was further shown that *Clock* and *Bmal1* expression in FS-PV cells participates in their maturation (Kobayashi et al., 2015). Mice mutants of *Clock* have altered nursing behavior that impacts cross-fostered wild-type pups. At postnatal day 14, these pups have reduced levels of brain serotonin, whose homeostasis during the early postnatal period is critical for normal emotional behavior in adulthood (Koizumi et al., 2013). Indeed, these animals have increased adult anxiety-related behavior. Furthermore, the photoperiod received during pre- and postnatal periods imprints the intrinsic electrical properties of serotonergic neurons of the dorsal raphe that can impact depression- and anxiety-related behavior later in life even after several subsequent photoperiod shifting (Green et al., 2015). Conversely, early-life adversity can change the hormonal milieu to increase the circulating levels of both glucocorticoids and pro-inflammatory cytokines that in turn deregulate circadian rhythms, leading to lasting epigenetic, physiological, and behavioral changes (Masri and Sassone-Corsi, 2010; Marco et al., 2016). Recent rodent and human studies have also revealed that PV expression and PNN accumulation have diurnal fluctuations in the adult (Pantazopoulos et al., 2020; Harkness et al., 2021), suggesting that circadian rhythms play a role in FS-PV function throughout life.

### Hormones

CPs can occur in parallel to developmental changes in hormone levels, along the adrenal, thyroid, and gonadal axes, which have the potential to calibrate neural circuits. Adverse experiences during early life can elicit stress responses from the hypothalamic-pituitary-adrenal (HPA) axis, which results in the release of corticosteroids from the adrenal glands. Exposure during CPs may result in maladaptation of the HPA-axis that will affect basal and stress-induced activity in adulthood (Van Bodegom et al., 2017). The effects of early-life stress are also manifested by changes in FS-PV cell development and maturation (Chen et al., 2018; Page et al., 2019; Nawreen et al., 2020; Vasistha et al., 2020), typically within the mPFC (Bueno-Fernandez et al., 2021), which has a negative feedback function on the HPA-axis (Van Bodegom et al., 2017). Implicated in behavioral and psychiatric disorders, the maturation of mPFC connectivity occurs in multiple CP windows from childhood through to adolescence enabling complex brain functions such as memory, cognition, decision making, social behaviors, and mood (for review, see Klune et al., 2021). Consequently, mPFC development is also impacted by gonadal hormones during adolescence, with ovarian hormones having been shown to drive both mouse pubescence and mPFC inhibitory activity independently of age (Piekarski et al., 2017). Progesterone is also required in early neonatal life for proper innervation of the mPFC that affects adult mouse behavioral impulses and cognitive flexibility (Willing and Wagner, 2016). In song birds, both estrogen and testosterone have been shown to impact inhibitory neuron development affecting either language acquisition and processing or song crystallization (Vahaba and Remage-Healey, 2018; Cornez et al., 2020). Along the hypothalamic-pituitary-thyroid (HPT) axis, it has been hypothesized that thyroid hormone levels could affect cholinergic activity that shapes FS-PV cell maturation (Batista and Hensch, 2019). Indeed, reduction of thyroid levels has been shown to reduce cortical PV expression in an age-dependent manner, suggesting there is a CP of thyroid hormone action (Uchida et al., 2021). Together or separately, these hormonal signals clearly have the potential to influence FS-PV cell maturation during multiple CP windows thereby affecting the etiology of behavioral and psychiatric disorders such as autism spectrum disorder, depression, and schizophrenia.

### Guidance Molecules and Morphogens

Implicated in axon guidance during development, semaphorins are chemorepulsive proteins that can be secreted and signal by binding to plexin receptors. In the postnatal rodent cortex, SEMA3A accumulates around FS-PV cells, owing to interaction with CSPGs, and colocalizes with various PNN components such as CSPGs and TNR (Dick et al., 2013; Vo et al., 2013; Nadanaka et al., 2020). It forms a complex with Plexin-A1 or Plexin-A4 receptors that is stabilized by Neuropilin-1 (NRP1; Lu et al., 2021). While *Sema3A* mRNA is found in neurons, there is no correlation between neuronal expression and PNN accumulation; instead, the choroid plexus has been proposed as a potential source *via* SEMA3A secretion into the CSF (de Winter et al., 2016). Regardless, SEMA3A accumulates in rodent visual cortex PNNs in an experience-dependent manner, and this accumulation participates in CP closure (Boggio et al., 2019). In the adult rat, blocking SEMA3A-NRP1 interaction can promote ocular dominance plasticity, showing that it is a required PNN-dependent component for maintaining low-plasticity (Boggio et al., 2019).

The OTX2 homeoprotein is expressed in the choroid plexus, secreted into the CSF, and accumulates within FS-PV cells owing to interactions with CSPGs in PNNs (Beurdeley et al., 2012; Spatazza et al., 2013). This signaling induces CP onset and mediates CP closure in primary visual and auditory cortices and in the mPFC (for review, see Di Nardo et al., 2020). Blocking OTX2-PNN interaction or sequestering OTX2 in the CSF in adult mice can promote ocular dominance plasticity (Beurdeley et al., 2012; Bernard et al., 2016), while overexpression of OTX2 in the choroid plexus can rescue plasticity-dependent anxiety-like behavior deficits regulated in the mPFC (Vincent et al., 2021). Upon accumulation within FS-PV cell PNNs, OTX2 can gain direct access to the cytoplasm and nucleus where it has been shown to regulate the translation of CSPGs and the transcription of proteins regulating oxidative stress response and DNA methylation (Hou et al., 2017; Sakai et al., 2017; Apulei et al., 2019). Thus, OTX2 participates in PNN growth, in cell metabolism, and in the regulation of FS-PV epigenetic states that directly impact FS-PV cell function. The accumulation of OTX2 in mPFC FS-PV cells has also been found to occur with diurnal fluctuations (Harkness et al., 2021), suggesting OTX2 may serve a role in coordinating diurnal changes in gene expression needed for optimal PV cell function during sleep and wakefulness.

## Insight on Epigenetic Mechanisms within FS-PV Cells

One of the final downstream outcomes of some non-cell-autonomous factors for FS-PV cell maturation may be to change chromatin states. DNA methylation and hydroxy-methylation are fast and precise systems of expression regulation, mainly repression (Greenberg and Bourc’his, 2019). In the brain, methylation occurs in both CpG islands and non-CpG contexts and may have diverse roles in CP regulation. DNA methyltransferase (DNMT) activity is required for experience-dependent methylation of key plasticity genes, and mediates ocular dominance shift after monocular deprivation during CP (Tognini et al., 2015). DNA methylation also acts as a marker for DNA binding proteins such as Methyl-CpG-binding protein 2 (MeCP2), a key methyl-DNA binding protein with a causal role in Rett syndrome. MeCP2 is also necessary for correct CP timing, as heterozygous mice show premature CP timing (Krishnan et al., 2015; Patrizi et al., 2020). This phenotype suggests accelerated FS-PV cell maturation, hypothesized to underly psychiatric disorders (Morishita et al., 2015). Furthermore, MeCP2 knock-out mice have increased basal and learning-induced PV expression, which is associated with CP dysfunction and anxiety (Morello et al., 2018). Histological analysis of MeCP2 distribution in FS-PV cells shows broad changes between plastic and non-plastic states, suggestive of global changes in chromatin structure. This distribution is influenced by the expression of GADD45b/g, which regulate the methylation of immediate-early genes implicated in plasticity (Apulei et al., 2019).

Histone post-translational modifications (hPTMs) impact chromatin structure and accessibility, which ultimately influence transcription regulation. The histone code is finely regulated and particularly difficult to study as it can include more than 100 different hPTMs that are often gene- and cell-specific (Millán-Zambrano et al., 2022). However, some experience-dependent marks have been linked to CP regulation, such as phosphorylated H3 and H3/H4 acetylation (Putignano et al., 2007). Functionally, broad pharmacological modulations of the enzymes responsible for acetylated hPTM maintenance such as histone deacetylases (HDAC) can restore plasticity in the adult in the primary visual cortex (Silingardi et al., 2010; Lennartsson et al., 2015). In the other direction, conditional HDAC2 knock-out specifically in FS-PV cells delays CP closure (Nott et al., 2015). Taken together, these results indicate that dynamic regulation of acetylation may be necessary to maintain a low-plastic state, but also that some degree of redundancy is possible.

Other potential epigenetic factors include microRNAs and long non-coding RNAs (lncRNA). In the primary visual cortex, Mir-29a expression increases with age independent of visual experience, yet is hypothesized to coordinate the expression of different epigenetic and ECM-related genes in FS-PV cells to facilitate juvenile CP or adult plasticity (Napoli et al., 2020). While lncRNAs are gaining attention in epigenetic research, only one study to date has investigated their involvement in CP visual cortex plasticity, highlighting specificity for cortical layer and plasticity state (Benoit et al., 2015). While their function in genomic regulation remains unknown, one hypothesis is that they regulate alternative splicing of transcripts in the maturing cortex. Further single-cell studies will be necessary to uncover the specificity and functions of these transcripts.

## Outlook

Non-cell-autonomous factors play a particular role in the regulation of brain CPs owing to the involvement of FS-PV cells, whose maturation includes epigenetic changes and complex changes in their local ECM, which in turn modify their response to these signaling factors. As a result, factors considered permissive in the juvenile brain, such as BDNF and OTX2, may be ignored or even be repressive in the adult brain. Indeed, perturbations of many of these non-cell-autonomous signals are sufficient to delay or accelerate CP, and some of them are crucial for CP onset or closure. Thus, the regulation of FS-PV cell activity is uniquely positioned by integrating information coming from systemic and circadian signals, from local and choroid-plexus-derived molecular signals, and from local and long-range synaptic inputs. Although not highlighted in this review, other factors, such as changes in inflammatory response due to early-life stress or changes in gut bacteria, have recently been shown to affect CPs (Smith et al., 2016; Ikezu et al., 2021; Lupori et al., 2022). Many questions remain, such as whether FS-PV cells act in concert with local glial cells by regulating the secretion of glial-derived factors. It is also unknown how the maturation-dependent changes in DNA methylation and chromatin conformation directly affect the function of FS-PV cells and their response to these factors.

An important point to consider is the implication of FS-PV cells not only in neurodevelopmental disorders, such as amblyopia but also in psychiatric diseases, due in part to their influence on global excitation/inhibition balance in cognitive development (Marín, 2012; Ferguson and Gao, 2018; Hensch and Quinlan, 2018). The re-opening of heightened plasticity in the adult may be a promising therapeutic strategy for these diseases. However, animal models exploring these strategies currently rely on either broad or non-specific methods, such as chondroitinase ABC, fluoxetine, and valproate, or on cell-specific strategies requiring virus-based gene therapy through stereotaxic injections (Dehorter and Pino, 2020; Nelson and Gabard-Durnam, 2020). The panoply of non-cell-autonomous factors for FS-PV cell maturation states include potential modulators of plasticity that may overcome plasticity brakes in adulthood. Improved understanding of the mechanisms involving these factors may lead to new molecules for the precise and gentle re-opening of CPs in therapeutic contexts.

## Author Contributions

All authors contributed equally to the writing of this review. All authors contributed to the article and approved the submitted version.

## Conflict of Interest

The authors declare that the research was conducted in the absence of any commercial or financial relationships that could be construed as a potential conflict of interest.

## Publisher’s Note

All claims expressed in this article are solely those of the authors and do not necessarily represent those of their affiliated organizations, or those of the publisher, the editors and the reviewers. Any product that may be evaluated in this article, or claim that may be made by its manufacturer, is not guaranteed or endorsed by the publisher.
